# Recapitulation of previously reported associations for type 2 diabetes and metabolic traits in the 126K East Asians

**DOI:** 10.5808/GI.2019.17.4.e48

**Published:** 2019-12-20

**Authors:** Ji-Young Choi, Hye-Mi Jang, Sohee Han, Mi Yeong Hwang, Bong-Jo Kim, Young Jin Kim

**Affiliations:** Division of Genome Research, Center for Genome Science, National Institute of Health, Osong Health Technology Administration Complex, Cheongju 28159, Korea

**Keywords:** genome-wide association study, glycemic index, phenotype, single nucleotide polymorphism, type 2 diabetes

## Abstract

Over the last decade, genome-wide association studies (GWASs) have provided an unprecedented amount of genetic variations that are associated with various phenotypes. However, previous GWAS were mostly conducted in European populations, and these biased results for non-Europeans may result in a significant reduction in risk prediction for non-Europeans. An issue with the early GWAS was the winner’s curse problem, which led to misleading results when constructing the polygenic risk scores (PRS). Therefore, more non-European population-based studies are needed to validate reported variants and improve genetic risk assessment across diverse populations. In this study, we validated 422 variants independently associated with glycemic indexes, liver enzymes, and type 2 diabetes in 125,872 samples from a Korean population, and further validated the results by assessing publicly available summary statistics from European GWAS (n = 898,130). Among the 422 independently associated variants, 284, 320, and 361 variants were replicated in Koreans, Europeans, and either one of the two populations. In addition, the effect sizes for Koreans and Europeans were moderately correlated (r = 0.33–0.68). However, 61 variants were not replicated in both Koreans and Europeans. Our findings provide valuable information on effect sizes and statistical significance, which is essential to improve the assessment of disease risk using PRS analysis.

## Introduction

Over the last decade, genome-wide association studies (GWASs) have served as an efficient tool for discovering genetic variants associated with various phenotypes [1]. Moreover, large-scale biobank data have enabled us to make rapid progress in identifying new variants [2-4]. Currently, the National Human Genome Research Institute European Bioinformatics Institute GWAS catalog contains numerous manually curated associated variants [5]. These cataloged variants can be used to construct a polygenic risk score (PRS), a summarized genetic risk of an individual, to profile the genetic risk of various diseases [6,7]. In a previous study, individuals with high PRS values (1.5%–8% of the population) showed a greater than three-fold risk of coronary artery disease, atrial fibrillation, type 2 diabetes (T2D), inflammatory bowel disease, and breast cancer [8].

Despite an abundance of scientific evidence on genetic associations, there are two significant limitations for generalizing genomics into clinical practice. First, genetic associations differ according to populations, and some associations are produced by the winner’s curse, the systematic overestimation of genetic effects in a particular population due to chance noise resulting in an unexpectedly low replication rate [9]. The other limitation is that a majority of the previous GWAS have been conducted in Europeans [7]. These studies may have biased results leading to a reduction in individual genetic risk prediction in non-Europeans [7]. Therefore, more non-European based studies are needed to validate the reported variants and improve genetic risk assessment across diverse populations [7].

**Fig. 1.**
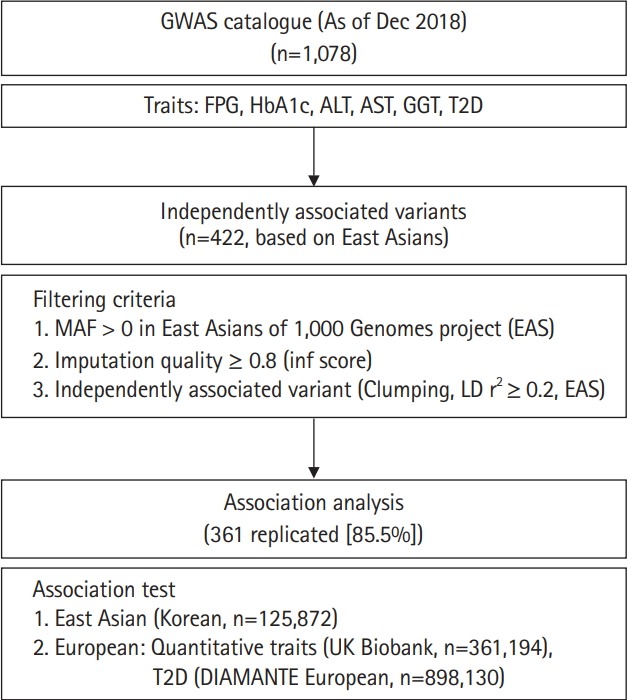
Overall analysis scheme.

In this study, we performed association tests on previously reported variants responsible for variations of glycemic indexes (fasting plasma glucose [FPG] and glycated hemoglobin [HbA1c]), T2D, and liver enzymes (alanine aminotransferase [ALT], aspartate aminotransferase [AST], and γ-glutamyl transferase [GGT]) in East Asians. Among the 1,078 associations known as of December 2018, 422 independently associated variants were analyzed using 125,872 samples from the Korean Genome and Epidemiology Study (KoGES) [10] genotyped with the Korea Biobank Array (KBA) [11]. In addition, association results from this study were compared to those of the UK biobank (n = 361,194 for biochemical traits) and European GWAS for T2D (n = 898,130). Furthermore, genetic effects were compared between East Asians and Europeans. The analysis flow of this study is summarized in Fig. 1.

## Methods

### Study subjects

The KoGES was initiated in 2001 to investigate genetic and environmental factors for complex traits. There were 211,725 participants (aged 40–70 years) recruited from three population-based cohorts, including the KoGES_Ansan and Ansung study, the KoGES_Health EXAminee (HEXA) study, and the KoGES_CardioVascular disease Associations Study (CAVAS) [10]. Participants were examined using epidemiological surveys, physical examinations, and laboratory tests. All participants provided informed consent. The study using the KoGES samples was approved by an institutional review board at the Korea National Institute of Health, Republic of Korea. The description of KoGES has been published previously [10].

### Phenotype measurements

Glycemic indexes (FPG and HbA1c) and liver enzymes (ALT, AST, and GGT) were measured. Participants with possible confounding factors (such as medication or therapy) were excluded from further analysis. The traits were inverse normal transformed to an approximate normal distribution [4]. T2D cases were defined based on the following criteria: diabetes diagnosis, T2D treatment, anti-diabetic treatment, FPG ≥7.0 mmol/L (126 mg/dL), plasma glucose 2 h after ingestion of 75 g oral glucose load ≥ 11.1 mmol/L (200 mg/dL, when available) or HbA1c ≥ 6.5% (when available). Controls were defined as having no history of T2D, FPG < 5.6 mmol/L, plasma glucose 2 h after ingestion of 75 g oral glucose load < 7.8 mmol/L (when available) and HbA1c < 6% (when available). There were 12,135 T2D cases and 94,636 controls.

### Genotyping and quality control

The KBA has been designed to contain tagging variants optimized for East Asians and functional variants selected from 2,576 sequenced Korean samples [11]. The detailed description of the design of the KBA project has been described previously [11]. Initially, 134,721 samples were genotyped using KBA. Genotypes from the samples were called by batches, with about 3,000 to 8,000 samples considered the recruitment site. Plink v1.9 was used for conducting quality control (QC) [12], which was performed according to the KBA QC and analysis protocol (http://www.koreanchip.org). Samples were excluded based on the following criteria: gender discrepancy, low call rate (<97%), excessive heterozygosity, outliers of the principal component analysis by using FlashPCA [13]. After sample QC, low-quality variants were removed if they were poorly clustered based on SNPolisher analysis results, with the missing rate > 5%, and the Hardy-Weinberg equilibrium failure p < 10^–6^. For the QC dataset, 2nd-degree relatives were removed from the dataset using KING v2 software [14]. Consequently, 125,872 samples remained for further analysis.

### Retrieving previously associated variants

The variants previously associated with any of the glycemic indexes, liver enzymes, or T2D were retrieved from a GWAS catalog database (https://www.ebi.ac.uk/gwas/). From this record, variants from a particular study with less than 1,000 samples were removed from further study to prevent possible false positives from winner’s curse of early GWAS efforts. As of December 31, 2018, there were 1,078 variants cataloged. Chromosomal positions were converted from hg38 to hg19 using LiftOver from the University of California Santa Cruz (UCSC) genome browser [15]. All variants located within 500 kb were clustered as a locus. Among 1,078 variants, variants were used if minor allele frequency (MAF) > 0 in 1,000 Genomes project phase 3 East Asians (1KG EAS) [16] and with imputation quality score (info ≥ 0.8) in this study. For selecting independent associated variants among the loci, clumping method was used for selecting the variants with the lowest p-value among correlated variants in a specific locus. To do this, pairwise linkage disequilibrium (LD) r^2^ among the loci was calculated using 504 samples of 1KG EAS data. As a threshold for clumping, LD r^2^ ≥ 0.2 in 1KG EAS was used. After filtering, 422 independently associated variants remained for further analysis (Fig. 1).

### Statistical analysis

The independently associated variants (n = 422) were imputed if they could not be directly genotyped. Pre-phasing based imputation was conducted using Eagle v2.3 for phasing and Impute v4 (https://jmarchini.org/software/) was used for genotype imputation [2,17]. For imputation, a merged reference panel of 2,504 samples of 1,000 Genomes project phase 3 and 397 Korean whole genome sequencing data was used as the reference panel [11]. Single variant associations were assessed through linear or logistic regression analysis based on alternative allele counts using EPACTS v3.4.6 (http://genome.sph.umich.edu/wiki/EPACTS) and adjusted for age, sex, and body mass index (for T2D). Scatter plots were generated using the R statistics program (version 3.4.4; https://www.r-project.org).

## Results

The overall analysis scheme is summarized in Fig. 1. As of December 2018, FPG, HbA1c, ALT, AST, GGT, and T2D associated variants were selected from a GWAS catalog database. To exclude possible false positives, studies with less than 1,000 samples were removed from further analysis. Initially, there were 1,078 variants associated with six traits of interest. Among 1,078 variants, variants were selected if MAF > 0 in 1KG EAS and with a high imputation quality score (info ≥ 0.8). In addition, variants were further refined to select independently associated variants by clumping, selecting the variants with the lowest p-value among correlated variants. For clumping, p-values of variants from GWAS catalog were used and LD threshold was set to r^2^ ≥ 0.2 using 1KG EAS. Consequently, 422 independently associated variants remained for further analysis. Of these, 216 (51.2%) were T2D variants. We also observed several loci that contained more than two independent variants. For example, there were five independently associated T2D variants at 6p21.33 (chr6:31136435–32685550) (Supplementary Table 1).

**Fig. 2.**
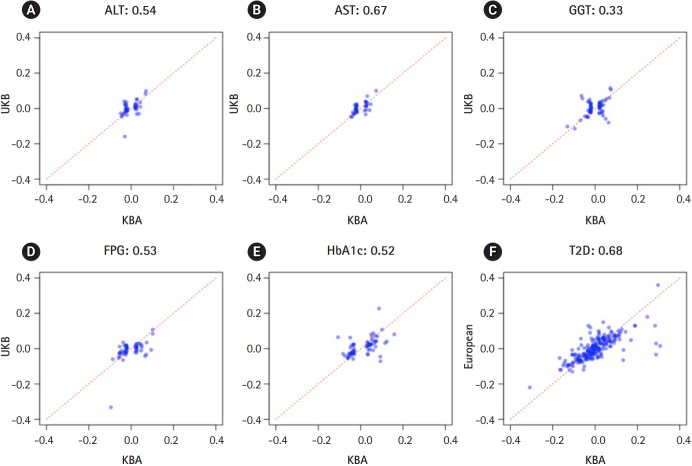
Effect sizes in East Asians and Europeans. X-axis represents effect sizes from Korea Biobank Array (KBA). Y-axis represents effect sizes from UK biobank association results. Red dotted line indicates a diagonal line.

The independently associated 422 variants were tested for an association with biochemical traits and T2D. The replication results are summarized in Table 1: 284 variants (67.3%) were associated with six traits (p ≤ 0.05) (Table 1, Supplementary Table 1). We further assessed the summary statistics of the European GWASs from the UK biobank (n = 361,194, downloaded from Neale lab, http://www.nealelab.is/) and DIAMANTE European (n = 898,130). In the European GWAS results, 320 variants were associated with six traits p ≤ 0.05) (Table 1). Based on the association results of either the KBA or European GWAS, 361 variants (85.5%) were found to be associated with six traits (p ≤ 0.05). However, 61 were not replicated in both the KBA and European GWASs (p > 0.05). Although the replication results varied by population due to differences in sample sizes and genetic architectures, effect sizes from the KBA and European studies were moderately correlated (Table 1, Fig. 2). The correlation coefficient (r) was 0.33–0.67 for liver enzymes, 0.52–0.53 for glycemic indexes, and 0.68 for T2D (Table 1). The 61 non-replicated variants showed genetic effect sizes close to zero in both the populations. Furthermore, the effect sizes of the KBA and European studies showed an increased correlation (r = 0.76) when the non-replicated variants were removed from the dataset. Notably, some of the variants were not available in the results of the European studies, possibly due to low allele frequency and technical problems arising from imputation analysis and association tests (Table 2).

## Discussion

In the current study, 422 biochemical traits and T2D variants were extensively validated in the 125,872 samples from either the Korean or European GWASs (n = 361,194; n = 898,130). Recently, Biobank Japan (BBJ) conducted a GWAS on biochemical traits and T2D in approximately 160,000 Japanese individuals [4,18]. However, the BBJ GWAS focused on the variants with statistical significance (p < 5 × 10^-8^). In our findings, there were multiple independent variants in a single locus. These independent variants would not be analyzed if a lead signal was selected for a given locus. Overall, we validated 361 of the 422 independently associated variants (p ≤ 0.05). In the present study, we also reported 61 non-replicated variants not found in either of the two populations, possibly due to the winner’s curse, technical problems, or because the study was conducted in a specific population other than East Asians or Europeans. However, further analyses are needed to investigate the reason for replication failures. Our findings provide valuable information regarding effect sizes and statistical significance, which is essential to improve the assessment of disease risk using PRS analysis.

Although these findings are valuable resources, our study is limited by a small sample size relative to the previously conducted European GWAS. For example, only 107 T2D variants (49.5%) were replicated in the KBA, whereas 155 variants (71.8%) were replicated in the results of European GWAS (n = 898,130). Given the highly correlated effect sizes across populations (r = 0.76 for replicated variants) (Fig. 2), this discrepancy is possibly due to the seven-fold smaller sample size of the KBA. A larger sample size or a meta-analysis through an international collaboration is required to perform a GWAS that is comparable to the European GWAS. The other limitation of this study is the use of summary statistics of the UK biobank downloaded from the Neale lab (http://www.nealelab.is/). The summary statistics from the Neale lab does not consider confounding factors such as medication and disease state that may influence biochemical traits. Therefore, a correlation analysis using the KBA and UK biobank results from the Neale lab would underestimate the correlation due to the possible confounding effects of the UK biobank results.

Recently, the utility of PRS in clinical practice has garnered considerable attention [6]. However, PRS may not accurately represent genetic risk at an individual level if unvalidated variants or only lead variants are used for constructing the PRS. In this context, our study provides valuable resources for constructing PRS in East Asians, particularly in a Korean population. However, validation efforts in a specific population should be conducted along with the continuous study of ethnically diverse populations.

## Figures and Tables

**Table 1.** Summary of replication results

Trait	No. of associations	EAS (p ≤ 0.05)	EUR (p ≤ 0.05)	EAS or EUR (p ≤ 0.05)	Not replicated	Correlation (r)
ALT	30	27	24	29	1	0.54
AST	29	27	20	27	2	0.67
GGT	55	50	48	53	2	0.33
FPG	54	41	40	47	7	0.53
HbA1c	38	32	33	37	1	0.52
T2D	216	107	155	168	48	0.68
Total	422	284	320	361	61	-

EAS, East Asians; EUR, European; ALT, alanine aminotransferase; AST, aspartate aminotransferase; GGT, γ-glutamyl transferase; FPG, fasting plasma glucose; HbA1c, glycated hemoglobin; T2D, type 2 diabetes.

**Table 2.** Number of variants not available in association results of Europeans

Trait	No. of associations	Not available	MAF = 0 (monomorphic)	MAF < 1%	MAF ≥ 1%	
ALT	30	2	1	1	0	
AST	29	5	3	2	0	
GGT	55	2	2	0	0	
FPG	54	3	1	1	1	
HbA1c	38	2	0	1	1	
T2D	216	7	1	1	5	
Total	422	21	8	6	7	

MAF, minor allele frequency; ALT, alanine aminotransferase; AST, aspartate aminotransferase; GGT, γ-glutamyl transferase; FPG, fasting plasma glucose; HbA1c, glycated hemoglobin; T2D, type 2 diabetes.
