# Supplementary material for: Recapitulation of previously reported associations for type 2 diabetes and metabolic traits in the 126K East Asians
Source: Genomics Inform. 2019 Dec 20;17(4):e48. doi: 10.5808/GI.2019.17.4.e48 (PMC6944053; doi:10.5808/GI.2019.17.4.e48)
Supplement: Supplementary file 1 [file gi-2019-17-4-e48-supplementary.pdf]

Supplementary Table 1. Association results of 422 independently associated variants

Chromosome and Position were based on hg19. Freq is effective allele frequency. P-value was marked as '0' if P-value was estimated as less than 1e-300.

						East Asian(KBA, n=125,872)				European (n=361,194~898,130)			
Trait	ID	Chromosome	Position(bp)	Effective allele	Other allele	Freq	Effect	StdErr	P.value	Freq	Effect	StdErr	P.value
ALT	10:101795361_T/A	10	101795361	A	T	0.1827	-0.0151	0.0056	6.54E-03	0.3985	-0.0300	0.0023	1.90E-39
ALT	10:113949664_T/C	10	113949664	C	T	0.6931	-0.0293	0.0047	4.21E-10	0.7276	0.0268	0.0025	1.52E-26
ALT	10:79680514_C/A	10	79680514	A	C	0.4987	0.0146	0.0043	6.86E-04	0.5962	0.0231	0.0023	3.09E-24
ALT	10:98460581_G/A	10	98460581	A	G	0.2806	0.0228	0.0049	3.18E-06	0.1861	0.0196	0.0029	1.13E-11
ALT	11:61573684_T/C	11	61573684	C	T	0.3044	-0.0246	0.0051	1.32E-06	0.3453	0.0086	0.0024	2.64E-04
ALT	11:93913692_T/C	11	93913692	C	T	0.1971	-0.0544	0.0054	7.76E-24	0.0740	0.0277	0.0043	9.07E-11
ALT	19:10347084_T/C	19	10347084	C	T	0.4779	-0.0285	0.0043	5.49E-11	0.8068	0.0158	0.0028	2.60E-08
ALT	19:41333284_T/C	19	41333284	C	T	0.4001	-0.0263	0.0045	4.52E-09	0.0699	0.0236	0.0044	8.48E-08
ALT	1:167156500_T/C	1	167156500	C	T	0.2637	-0.0003	0.0049	9.49E-01	0.3732	0.0022	0.0023	3.51E-01
ALT	21:30126482_A/G	21	30126482	G	A	0.2499	0.0198	0.0050	7.63E-05	0.1430	-0.0168	0.0032	1.42E-07
ALT	22:44324727_C/G	22	44324727	G	C	0.4161	0.0774	0.0043	2.52E-71	0.2158	-0.0992	0.0027	6.36E-294
ALT	2:169870295_C/A	2	169870295	A	C	0.3315	0.0412	0.0046	2.87E-19	0.0553	0.0344	0.0049	1.83E-12
ALT	2:211540507_C/A	2	211540507	A	C	0.1782	0.0381	0.0056	8.73E-12	0.3157	0.0096	0.0024	7.01E-05
ALT	2:233520254_A/T	2	233520254	T	A	0.2613	0.0407	0.0050	3.06E-16	0.6002	-0.0294	0.0023	1.57E-37
ALT	2:37149498_T/C	2	37149498	C	T	0.6426	-0.0075	0.0046	1.02E-01	0.4696	0.0069	0.0022	2.07E-03
ALT	3:135926622_G/T	3	135926622	T	G	0.8742	0.0252	0.0065	9.51E-05	0.7734	0.0314	0.0027	6.34E-32
ALT	3:149212268_G/A	3	149212268	A	G	0.0232	0.0540	0.0143	1.58E-04	0.0053	0.0862	0.0158	4.56E-08
ALT	4:146821410_C/A	4	146821410	A	C	0.4185	0.0326	0.0043	6.09E-14	0.1585	0.0527	0.0031	1.59E-66
ALT	4:77416627_A/G	4	77416627	G	A	0.5526	-0.0305	0.0043	1.44E-12	0.6467	0.0181	0.0023	1.05E-14
ALT	4:88176030_C/A	4	88176030	A	C	0.3229	-0.0500	0.0046	2.22E-27	0.2327	-0.0376	0.0026	6.78E-46
ALT	5:56087899_C/T	5	56087899	T	C	0.1434	-0.0170	0.0062	5.67E-03	0.0716	-0.0054	0.0044	2.17E-01
ALT	6:33860843_G/T	6	33860843	T	G	0.0479	0.0344	0.0100	5.75E-04	0.0566	0.0028	0.0049	5.69E-01
ALT	7:28179258_C/A	7	28179258	A	C	0.3782	0.0208	0.0045	2.92E-06	0.0906	0.0218	0.0039	2.68E-08
ALT	7:55937441_T/A	7	55937441	A	T	0.4044	0.0201	0.0044	5.37E-06	0.4819	0.0044	0.0023	5.68E-02
ALT	7:87079406_T/A	7	87079406	A	T	0.2900	-0.0314	0.0047	2.86E-11	0.1802	-0.0310	0.0029	1.39E-26
ALT	8:126490972_A/T	8	126490972	T	A	0.5583	-0.0347	0.0043	1.07E-15	0.4651	0.0400	0.0023	7.62E-71
ALT	9:117140082_T/C	9	117140082	C	T	0.4905	-0.0399	0.0044	1.04E-19	0.4872	0.0468	0.0023	1.80E-96
ALT	9:6665010_C/T	9	6665010	T	C	0.1155	0.0010	0.0067	8.78E-01	0.1390	-0.0167	0.0032	2.26E-07
ALT	12:111718231_C/A	12	111718231	A	C	0.1796	-0.0424	0.0057	1.02E-13	NA	NA	NA	NA
ALT	12:112817783_T/A	12	112817783	A	T	0.1676	-0.0529	0.0057	2.22E-20	NA	NA	NA	NA
AST	10:100240374_C/T	10	100240374	T	C	0.0692	-0.0474	0.0088	6.63E-08	0.1190	-0.0059	0.0036	9.84E-02
AST	10:101132282_A/C	10	101132282	C	A	0.3561	0.0397	0.0047	2.32E-17	0.7291	-0.0331	0.0026	1.21E-37
AST	10:101520941_G/A	10	101520941	A	G	0.0181	-0.0312	0.0163	5.48E-02	0.1151	-0.0071	0.0036	5.11E-02
AST	10:18222342_T/C	10	18222342	C	T	0.2941	0.0502	0.0048	1.15E-25	0.3241	-0.0243	0.0025	1.01E-22

AST	10:18510448_G/A	10	18510448	A	G	0.3855	0.0373	0.0044	3.75E-17	0.7231	0.0141	0.0026	3.63E-08
AST	11:93913692_T/C	11	93913692	C	T	0.1971	-0.0405	0.0054	6.18E-14	0.0740	0.0207	0.0044	2.27E-06
AST	16:58764855_C/A	16	58764855	A	C	0.4366	0.0202	0.0044	3.98E-06	0.0597	0.0379	0.0048	5.10E-15
AST	19:41333284_T/C	19	41333284	C	T	0.4001	-0.0154	0.0045	5.84E-04	0.0698	0.0060	0.0045	1.83E-01
AST	19:50012574_G/A	19	50012574	A	G	0.1608	0.0175	0.0061	3.78E-03	0.1627	0.0143	0.0031	4.31E-06
AST	1:110470764_G/A	1	110470764	A	G	0.3193	-0.0448	0.0048	4.20E-21	0.1495	-0.0469	0.0032	1.68E-48
AST	1:183802259_T/C	1	183802259	C	T	0.2874	0.0223	0.0047	2.23E-06	0.2019	-0.0359	0.0029	2.32E-36
AST	22:44324730_C/T	22	44324730	T	C	0.4157	0.0777	0.0043	8.57E-72	0.2159	0.1002	0.0028	4.89E-286
AST	2:169870295_C/A	2	169870295	A	C	0.3315	0.0252	0.0046	3.79E-08	0.0553	0.0236	0.0050	2.24E-06
AST	2:233503989_G/A	2	233503989	A	G	0.2291	0.0280	0.0053	1.01E-07	0.4491	0.0127	0.0023	4.05E-08
AST	4:146821410_C/A	4	146821410	A	C	0.4185	0.0225	0.0043	2.16E-07	0.1585	0.0401	0.0031	1.68E-37
AST	4:88176030_C/A	4	88176030	A	C	0.3229	-0.0344	0.0046	8.30E-14	0.2327	-0.0317	0.0027	1.29E-31
AST	5:39424628_A/C	5	39424628	C	A	0.3919	0.0286	0.0046	6.76E-10	0.6562	-0.0077	0.0024	1.48E-03
AST	5:72395189_G/A	5	72395189	A	G	0.3837	0.0242	0.0045	5.86E-08	0.5233	0.0337	0.0023	5.85E-49
AST	6:135419631_A/G	6	135419631	G	A	0.3270	-0.0244	0.0046	1.07E-07	0.2605	0.0222	0.0026	2.44E-17
AST	6:32191339_C/T	6	32191339	T	C	0.1478	0.0486	0.0060	5.54E-16	0.0778	-0.0093	0.0043	3.03E-02
AST	6:33076268_T/C	6	33076268	C	T	0.6164	0.0192	0.0044	1.24E-05	0.4694	-0.0138	0.0023	1.93E-09
AST	7:50258479_A/T	7	50258479	T	A	0.3404	-0.0178	0.0046	1.12E-04	0.3107	-0.0015	0.0025	5.39E-01
AST	9:117134191_C/T	9	117134191	T	C	0.5028	-0.0227	0.0044	2.11E-07	0.4932	-0.0278	0.0023	1.20E-33
AST	9:33117965_C/T	9	33117965	T	C	0.6949	-0.0273	0.0047	8.12E-09	0.3647	-0.0302	0.0024	1.61E-36
AST	12:111718231_C/A	12	111718231	A	C	0.1796	-0.0694	0.0057	4.22E-34	NA	NA	NA	NA
AST	7:80174361_T/C	7	80174361	C	T	0.0749	-0.0550	0.0087	2.50E-10	NA	NA	NA	NA
AST	10:17735719_T/C	10	17735719	C	T	0.0154	0.0002	0.0179	9.92E-01	NA	NA	NA	NA
AST	4:79619199_C/A	4	79619199	A	C	0.1378	-0.0374	0.0063	3.97E-09	NA	NA	NA	NA
AST	12:110069190_G/T	12	110069190	T	G	0.0441	-0.0613	0.0106	6.44E-09	NA	NA	NA	NA
FPG	10:113042093_T/G	10	113042093	G	T	0.9067	0.0377	0.0073	2.52E-07	0.8758	-0.0386	0.0038	1.19E-24
FPG	10:114758349_C/T	10	114758349	T	C	0.0276	-0.0125	0.0132	3.44E-01	0.2901	0.0536	0.0027	1.39E-85
FPG	11:2735534_C/G	11	2735534	G	C	0.7684	0.0194	0.0053	2.65E-04	0.3194	-0.0129	0.0027	1.65E-06
FPG	11:2857233_G/A	11	2857233	A	G	0.3925	-0.0576	0.0045	3.58E-37	0.0505	-0.0224	0.0057	9.02E-05
FPG	11:45873091_A/C	11	45873091	C	A	0.2075	-0.0240	0.0053	5.55E-06	0.5203	-0.0033	0.0025	1.90E-01
FPG	11:46300722_G/A	11	46300722	A	G	0.0782	-0.0060	0.0082	4.63E-01	0.5480	-0.0065	0.0025	1.10E-02
FPG	11:47336320_A/T	11	47336320	T	A	0.0216	-0.0120	0.0149	4.24E-01	0.2760	0.0312	0.0028	3.14E-29
FPG	11:61571478_T/C	11	61571478	C	T	0.3042	-0.0486	0.0051	8.47E-22	0.3457	0.0196	0.0026	5.87E-14
FPG	11:72432985_G/A	11	72432985	A	G	0.0609	-0.0578	0.0088	6.24E-11	0.1553	-0.0240	0.0034	2.30E-12
FPG	11:8243798_A/G	11	8243798	G	A	0.1026	-0.0175	0.0070	1.28E-02	0.4621	0.0112	0.0025	6.93E-06
FPG	11:92708710_C/G	11	92708710	G	C	0.4318	0.1021	0.0043	3.07E-125	0.2750	0.0101	0.0028	2.81E-04
FPG	13:28499962_G/A	13	28499962	A	G	0.4583	0.0439	0.0043	2.38E-24	0.2190	0.0295	0.0030	1.06E-22
FPG	13:28510712_T/C	13	28510712	C	T	0.4243	0.0136	0.0043	1.63E-03	0.2924	-0.0149	0.0027	4.95E-08

FPG	14:90034972_G/A	14	90034972	A	G	0.1181	-0.0269	0.0066	4.98E-05	0.6052	-0.0215	0.0026	3.50E-17
FPG	15:62396942_G/A	15	62396942	A	G	0.4600	-0.0538	0.0043	1.90E-36	0.4287	0.0115	0.0025	4.37E-06
FPG	15:62433962_A/G	15	62433962	G	A	0.3507	-0.0043	0.0046	3.41E-01	0.3838	-0.0089	0.0026	5.42E-04
FPG	15:99258710_A/T	15	99258710	T	A	0.5247	-0.0270	0.0043	3.29E-10	0.0035	0.0084	0.0226	7.10E-01
FPG	15:99258710_A/T	15	99258710	T	A	0.5247	-0.0270	0.0043	3.29E-10	0.6128	0.0098	0.0026	1.23E-04
FPG	1:214159256_T/C	1	214159256	C	T	0.3671	0.0219	0.0044	7.25E-07	0.5715	-0.0283	0.0025	2.08E-29
FPG	20:10434530_T/A	20	10434530	A	T	0.2078	-0.0020	0.0054	7.09E-01	0.0555	-0.0045	0.0054	4.04E-01
FPG	20:22592430_G/T	20	22592430	T	G	0.8372	0.0735	0.0058	6.06E-37	0.7848	0.0068	0.0030	2.38E-02
FPG	2:169763148_T/C	2	169763148	C	T	0.9724	0.1314	0.0129	2.93E-24	0.7011	-0.1555	0.0027	0.00E+00
FPG	2:169764491_T/C	2	169764491	C	T	0.0205	-0.2567	0.0155	1.61E-61	0.0017	0.2766	0.0301	4.50E-20
FPG	2:169767148_T/C	2	169767148	C	T	0.3538	0.1024	0.0045	7.24E-115	0.0128	-0.1032	0.0111	1.16E-20
FPG	2:173546313_G/A	2	173546313	A	G	0.5704	0.0476	0.0044	6.68E-28	0.0276	0.0036	0.0076	6.38E-01
FPG	2:27152874_C/T	2	27152874	T	C	0.1900	0.0188	0.0054	5.41E-04	0.2455	0.0214	0.0029	1.24E-13
FPG	2:27741237_T/C	2	27741237	C	T	0.4635	0.0716	0.0043	5.10E-63	0.6183	-0.0385	0.0026	2.82E-51
FPG	2:27995781_A/C	2	27995781	C	A	0.4146	-0.0192	0.0043	8.87E-06	0.7357	0.0156	0.0028	3.11E-08
FPG	2:45192080_G/C	2	45192080	C	G	0.3741	0.0776	0.0045	1.84E-67	0.1703	0.0345	0.0033	4.56E-25
FPG	3:170717521_T/A	3	170717521	A	T	0.0052	-0.0544	0.0289	6.01E-02	0.1245	-0.0690	0.0038	5.06E-75
FPG	3:170735099_G/A	3	170735099	A	G	0.1921	-0.0280	0.0054	2.54E-07	0.2569	-0.0581	0.0029	1.42E-92
FPG	3:185507299_A/T	3	185507299	T	A	0.3145	0.0257	0.0046	2.53E-08	0.3141	-0.0119	0.0027	9.46E-06
FPG	3:30346968_A/G	3	30346968	G	A	0.1233	-0.0070	0.0065	2.83E-01	0.1891	-0.0017	0.0032	6.01E-01
FPG	4:121801790_G/A	4	121801790	A	G	0.3821	-0.0025	0.0045	5.74E-01	0.1976	0.0048	0.0031	1.28E-01
FPG	5:129943319_T/C	5	129943319	C	T	0.3544	0.0086	0.0045	5.50E-02	0.1971	0.0031	0.0031	3.18E-01
FPG	5:79480620_T/C	5	79480620	C	T	0.8509	-0.0004	0.0062	9.52E-01	0.5607	-0.0014	0.0025	5.84E-01
FPG	5:95542726_C/A	5	95542726	A	C	0.2989	-0.0145	0.0047	1.91E-03	0.3069	-0.0258	0.0027	9.69E-22
FPG	6:20667382_T/C	6	20667382	C	T	0.4800	0.0472	0.0043	7.45E-28	0.3109	-0.0207	0.0027	1.51E-14
FPG	6:39033595_G/A	6	39033595	A	G	0.2082	-0.0479	0.0053	1.14E-19	0.0029	-0.0296	0.0236	2.10E-01
FPG	6:7240577_A/T	6	7240577	T	A	0.2103	-0.0016	0.0053	7.71E-01	0.3483	-0.0196	0.0026	6.86E-14
FPG	7:14898282_C/T	7	14898282	T	C	0.4191	0.0617	0.0044	6.82E-44	0.1732	0.0366	0.0033	1.25E-28
FPG	7:15064309_G/T	7	15064309	T	G	0.6740	0.0665	0.0045	1.21E-48	0.5478	0.0419	0.0025	2.79E-63
FPG	7:44205906_A/C	7	44205906	C	A	0.4918	0.0427	0.0043	1.71E-23	0.3723	-0.0560	0.0026	3.85E-105
FPG	7:44235668_G/A	7	44235668	A	G	0.2163	0.1010	0.0052	7.68E-85	0.1777	0.1078	0.0032	1.02E-241
FPG	7:50751090_T/C	7	50751090	C	T	0.8987	-0.0380	0.0071	6.95E-08	0.7727	0.0212	0.0030	7.91E-13
FPG	8:118184783_C/T	8	118184783	T	C	0.3956	-0.0641	0.0044	1.02E-48	0.3112	-0.0463	0.0027	1.04E-66
FPG	8:118549376_T/C	8	118549376	C	T	0.0414	0.0115	0.0107	2.83E-01	0.0670	0.0077	0.0050	1.23E-01
FPG	8:40484239_T/C	8	40484239	C	T	0.7221	-0.0062	0.0048	1.95E-01	0.8819	-0.0059	0.0039	1.28E-01
FPG	8:9183596_A/G	8	9183596	G	A	0.9895	-0.0624	0.0212	3.28E-03	0.9082	0.0444	0.0043	8.83E-25
FPG	9:22132698_T/C	9	22132698	C	T	0.4374	-0.0651	0.0043	1.44E-51	0.1768	0.0303	0.0033	1.63E-20
FPG	9:4289050_C/A	9	4289050	A	C	0.4051	0.0472	0.0044	2.41E-27	0.4780	0.0190	0.0025	2.39E-14

FPG	12:112645401_G/A	12	112645401	A	G	0.1424	-0.0968	0.0061	2.89E-57	NA	NA	NA	NA
FPG	9:622523_G/T	9	622523	T	G	0.1690	0.0701	0.0057	5.92E-35	NA	NA	NA	NA
FPG	12:121938185_C/G	12	121938185	G	C	0.2351	-0.0184	0.0052	3.67E-04	NA	NA	NA	NA
GGT	10:79636161_C/T	10	79636161	T	C	0.2108	0.0573	0.0054	4.11E-26	0.3427	0.0505	0.0024	1.70E-102
GGT	10:79688208_A/G	10	79688208	G	A	0.7670	0.0464	0.0053	1.85E-18	0.6183	-0.0510	0.0023	2.51E-110
GGT	11:34764919_C/A	11	34764919	A	C	0.7438	0.0018	0.0051	7.27E-01	0.1583	-0.0072	0.0030	1.86E-02
GGT	11:62199817_T/C	11	62199817	C	T	0.2951	-0.0346	0.0049	1.95E-12	0.1783	0.0448	0.0029	1.26E-53
GGT	12:111515020_G/C	12	111515020	C	G	0.1151	0.0864	0.0071	4.40E-34	0.0675	0.0537	0.0044	1.05E-33
GGT	12:121420260_A/G	12	121420260	G	A	0.5254	0.0934	0.0044	1.81E-98	0.6162	-0.1134	0.0023	0.00E+00
GGT	12:53274674_G/T	12	53274674	T	G	0.2081	0.0138	0.0055	1.18E-02	0.0523	0.0122	0.0050	1.41E-02
GGT	14:102994549_C/A	14	102994549	A	C	0.0237	-0.0070	0.0145	6.28E-01	0.0938	0.0051	0.0038	1.88E-01
GGT	14:103550388_G/C	14	103550388	C	G	0.5515	0.0195	0.0045	1.73E-05	0.7644	0.0022	0.0026	4.02E-01
GGT	14:103575070_A/G	14	103575070	G	A	0.6467	0.0543	0.0047	2.20E-30	0.4525	-0.0926	0.0023	0.00E+00
GGT	15:39932972_A/G	15	39932972	G	A	0.3814	0.0128	0.0045	4.71E-03	0.2828	-0.0057	0.0025	2.31E-02
GGT	15:60883281_C/A	15	60883281	A	C	0.9119	0.0404	0.0078	2.36E-07	0.6148	0.0597	0.0023	3.11E-151
GGT	15:73986264_G/T	15	73986264	T	G	0.4469	0.0142	0.0045	1.41E-03	0.4598	0.0324	0.0022	7.20E-48
GGT	16:80497601_C/A	16	80497601	A	C	0.3885	0.0255	0.0045	1.60E-08	0.2694	0.0501	0.0025	1.01E-89
GGT	17:70098161_G/C	17	70098161	C	G	0.8399	0.0152	0.0060	1.14E-02	0.6726	0.0274	0.0024	4.67E-31
GGT	17:73175294_T/C	17	73175294	C	T	0.6248	0.0067	0.0046	1.41E-01	0.2796	-0.0088	0.0025	3.73E-04
GGT	18:56086820_C/T	18	56086820	T	C	0.8277	-0.0666	0.0059	8.11E-30	0.7971	-0.0720	0.0028	1.30E-149
GGT	1:111684276_A/G	1	111684276	G	A	0.1831	-0.0151	0.0057	8.45E-03	0.1086	0.0296	0.0036	9.67E-17
GGT	1:154086512_G/A	1	154086512	A	G	0.4450	0.0129	0.0045	4.28E-03	0.3222	0.0108	0.0024	5.80E-06
GGT	1:155126948_T/C	1	155126948	C	T	0.8719	-0.0229	0.0066	5.64E-04	0.4053	0.0530	0.0023	1.37E-121
GGT	1:16510894_C/T	1	16510894	T	C	0.7704	0.0535	0.0054	2.10E-23	0.5804	0.0800	0.0023	8.46E-277
GGT	1:178513411_C/T	1	178513411	T	C	0.4939	0.0132	0.0044	2.86E-03	0.5344	0.0270	0.0022	9.51E-34
GGT	1:200261014_C/A	1	200261014	A	C	0.4397	0.0252	0.0045	3.11E-08	0.0018	-0.0117	0.0275	6.72E-01
GGT	1:23376541_T/G	1	23376541	G	T	0.4998	0.0095	0.0045	3.72E-02	0.1890	-0.0082	0.0029	4.32E-03
GGT	1:89146234_C/A	1	89146234	A	C	0.5364	0.0216	0.0044	1.02E-06	0.5549	0.0261	0.0022	9.39E-32
GGT	21:30555154_G/A	21	30555154	A	G	0.1974	0.0195	0.0057	5.82E-04	0.0099	0.0262	0.0113	2.04E-02
GGT	21:46271452_C/T	21	46271452	T	C	0.4401	-0.0140	0.0045	1.73E-03	0.5317	-0.0235	0.0022	5.45E-26
GGT	22:18439958_C/T	22	18439958	T	C	0.0242	-0.0390	0.0143	6.36E-03	0.2399	-0.0733	0.0026	5.67E-175
GGT	22:24256894_T/C	22	24256894	C	T	0.4594	0.0302	0.0045	1.49E-11	0.2642	-0.0290	0.0025	1.62E-30
GGT	22:24994296_T/C	22	24994296	C	T	0.3479	0.1835	0.0046	0.00E+00	0.3527	-0.1949	0.0023	0.00E+00
GGT	22:38971950_T/C	22	38971950	C	T	0.5026	0.0125	0.0044	4.84E-03	0.3691	-0.0227	0.0023	6.49E-23
GGT	2:169834370_G/A	2	169834370	A	G	0.3276	0.0302	0.0048	3.89E-10	0.0398	0.0440	0.0057	1.66E-14
GGT	2:192057406_A/C	2	192057406	C	A	0.6519	-0.0298	0.0047	1.69E-10	0.6502	0.0161	0.0023	4.56E-12
GGT	2:233520254_A/T	2	233520254	T	A	0.2613	0.0294	0.0051	1.01E-08	0.6002	-0.0272	0.0023	9.21E-33
GGT	2:27730940_T/C	2	27730940	C	T	0.4517	-0.0590	0.0044	2.48E-40	0.6069	0.0564	0.0023	4.00E-136

GGT	2:66073552_G/A	2	66073552	A	G	0.1045	-0.0056	0.0073	4.44E-01	0.1374	-0.0018	0.0032	5.84E-01
GGT	3:149186927_A/G	3	149186927	G	A	0.0305	-0.0697	0.0133	1.75E-07	0.0056	0.0420	0.0158	7.90E-03
GGT	3:149210443_T/C	3	149210443	C	T	0.4979	0.0332	0.0045	1.14E-13	0.5944	-0.0125	0.0023	3.64E-08
GGT	3:170725542_G/A	3	170725542	A	G	0.0052	0.0098	0.0301	7.46E-01	0.1238	0.0588	0.0034	3.42E-68
GGT	3:4909440_T/C	3	4909440	C	T	0.5269	0.0287	0.0045	2.71E-10	0.1093	-0.0204	0.0036	1.01E-08
GGT	4:146821410_C/A	4	146821410	A	C	0.4185	0.0803	0.0045	3.23E-72	0.1585	0.1057	0.0030	7.17E-266
GGT	4:3446883_A/G	4	3446883	G	A	0.4635	-0.0213	0.0045	1.75E-06	0.2517	0.0206	0.0026	2.35E-15
GGT	5:156743083_G/C	5	156743083	C	G	0.2628	-0.0261	0.0050	1.88E-07	0.0537	-0.0499	0.0049	3.69E-24
GGT	5:31020521_A/G	5	31020521	G	A	0.2570	-0.0205	0.0051	4.88E-05	0.2831	0.0539	0.0025	9.90E-106
GGT	5:52193125_A/G	5	52193125	G	A	0.0675	0.0729	0.0088	1.74E-16	0.0745	-0.0704	0.0042	4.11E-62
GGT	6:158484904_G/C	6	158484904	C	G	0.0215	-0.1109	0.0152	2.69E-13	0.0000	-21.7730	13.8040	1.15E-01
GGT	6:53903152_G/A	6	53903152	A	G	0.5057	-0.0230	0.0044	2.36E-07	0.6894	-0.0388	0.0024	1.04E-58
GGT	7:116438511_G/T	7	116438511	T	G	0.8673	-0.0151	0.0067	2.35E-02	0.6734	-0.0256	0.0024	3.39E-27
GGT	7:26022414_A/C	7	26022414	C	A	0.7044	0.0410	0.0049	7.19E-17	0.7933	-0.0412	0.0029	2.01E-47
GGT	7:73042614_A/G	7	73042614	G	A	0.1133	-0.0671	0.0070	8.01E-22	0.2785	0.0411	0.0025	2.08E-61
GGT	7:97826232_G/A	7	97826232	A	G	0.4451	-0.0177	0.0045	7.17E-05	0.3763	-0.0049	0.0023	3.20E-02
GGT	8:126482077_A/G	8	126482077	G	A	0.5697	-0.0153	0.0045	5.89E-04	0.5057	0.0305	0.0022	5.96E-43
GGT	9:131466489_G/T	9	131466489	T	G	0.0714	-0.0486	0.0085	1.16E-08	0.1457	-0.0442	0.0032	8.69E-45
GGT	12:112645401_G/A	12	112645401	A	G	0.1424	-0.1915	0.0063	3.59E-205	NA	NA	NA	NA
GGT	14:103814575_C/T	14	103814575	T	C	0.0185	-0.0561	0.0167	7.98E-04	NA	NA	NA	NA
HbA1c	10:114754088_T/C	10	114754088	C	T	0.0289	-0.0118	0.0189	5.32E-01	0.3090	-0.0646	0.0025	5.02E-148
HbA1c	10:12253597_G/A	10	12253597	A	G	0.4528	0.0414	0.0063	6.90E-11	0.2396	0.0287	0.0027	1.53E-26
HbA1c	10:71099913_T/C	10	71099913	C	T	0.2890	-0.0161	0.0069	1.90E-02	0.5125	0.0832	0.0023	2.12E-287
HbA1c	10:94466439_A/G	10	94466439	G	A	0.8518	-0.0433	0.0088	8.94E-07	0.4346	0.0274	0.0023	2.93E-32
HbA1c	11:199256_G/A	11	199256	A	G	0.5408	0.0450	0.0065	4.20E-12	0.1871	0.0217	0.0030	3.11E-13
HbA1c	11:61619829_C/A	11	61619829	A	C	0.6584	0.0438	0.0071	6.94E-10	0.6253	0.0213	0.0024	3.61E-19
HbA1c	11:92673828_C/T	11	92673828	T	C	0.4204	0.0502	0.0063	1.52E-15	0.2894	0.0517	0.0025	1.25E-92
HbA1c	12:48409054_C/A	12	48409054	A	C	0.1107	-0.0160	0.0100	1.08E-01	0.4716	-0.0618	0.0023	2.34E-158
HbA1c	13:113331868_A/G	13	113331868	G	A	0.0664	0.0127	0.0128	3.22E-01	0.1421	-0.0518	0.0033	9.46E-56
HbA1c	15:77799657_A/G	15	77799657	G	A	0.4312	0.0267	0.0063	2.60E-05	0.6096	-0.0224	0.0024	3.32E-21
HbA1c	16:88858646_T/G	16	88858646	G	T	0.6455	0.0972	0.0069	7.37E-45	0.5812	-0.0556	0.0024	1.56E-121
HbA1c	17:42241929_T/G	17	42241929	G	T	0.0774	-0.0178	0.0118	1.30E-01	0.0363	0.0043	0.0062	4.89E-01
HbA1c	17:76121864_A/G	17	76121864	G	A	0.2275	0.1298	0.0074	6.64E-69	0.2183	-0.0800	0.0028	2.87E-182
HbA1c	17:76137477_T/G	17	76137477	G	T	0.1793	0.0370	0.0081	5.17E-06	0.0986	-0.0059	0.0039	1.25E-01
HbA1c	17:80685533_C/T	17	80685533	T	C	0.4746	0.0901	0.0062	2.03E-47	0.3121	0.0691	0.0025	1.06E-170
HbA1c	19:17232499_C/T	19	17232499	T	C	0.3744	-0.0839	0.0065	8.52E-38	0.2703	-0.0286	0.0026	4.92E-28
HbA1c	19:46159986_C/T	19	46159986	T	C	0.4688	0.0440	0.0066	2.93E-11	0.1085	0.0259	0.0037	3.34E-12
HbA1c	1:156255456_G/A	1	156255456	A	G	0.2320	-0.0994	0.0073	6.76E-42	0.2933	0.0037	0.0025	1.40E-01

HbA1c	1:158585415_C/T	1	158585415	T	C	0.4154	0.0621	0.0063	1.05E-22	0.2660	0.0801	0.0026	7.01E-209
HbA1c	22:37462936_A/G	22	37462936	G	A	0.4891	-0.0611	0.0062	9.11E-23	0.5615	0.0625	0.0023	4.07E-159
HbA1c	2:169767148_T/C	2	169767148	C	T	0.3538	0.0712	0.0066	3.15E-27	0.0128	-0.0870	0.0102	1.89E-17
HbA1c	2:169791438_A/G	2	169791438	G	A	0.9875	0.0530	0.0288	6.60E-02	0.6455	-0.0901	0.0024	4.68E-308
HbA1c	2:45192080_G/C	2	45192080	C	G	0.3741	0.0651	0.0065	2.03E-23	0.1701	0.0295	0.0031	1.28E-21
HbA1c	3:170732599_G/A	3	170732599	A	G	0.1910	-0.0394	0.0079	6.49E-07	0.2820	-0.0414	0.0026	5.94E-59
HbA1c	3:185518921_G/A	3	185518921	A	G	0.2927	0.0278	0.0069	5.10E-05	0.3134	0.0217	0.0025	2.31E-18
HbA1c	6:135418916_A/G	6	135418916	G	A	0.3261	-0.0828	0.0067	2.94E-35	0.2620	0.0422	0.0026	1.50E-58
HbA1c	6:20675792_C/A	6	20675792	A	C	0.4644	0.0637	0.0063	5.02E-24	0.2614	0.0435	0.0026	6.96E-62
HbA1c	7:127841626_C/A	7	127841626	A	C	0.1074	0.0418	0.0103	4.88E-05	0.2758	-0.0054	0.0026	3.76E-02
HbA1c	7:44229068_C/T	7	44229068	T	C	0.1822	0.1166	0.0081	1.66E-47	0.1784	0.1077	0.0030	2.13E-282
HbA1c	8:118184783_C/T	8	118184783	T	C	0.3956	-0.0590	0.0064	2.61E-20	0.3110	-0.0562	0.0025	2.36E-113
HbA1c	8:41549194_T/C	8	41549194	C	T	0.0512	0.0226	0.0143	1.14E-01	0.0379	0.1348	0.0060	4.47E-111
HbA1c	8:41630405_G/A	8	41630405	A	G	0.5199	0.0698	0.0062	4.31E-29	0.2353	0.0731	0.0027	5.93E-161
HbA1c	9:110536932_G/A	9	110536932	A	G	0.8165	-0.0500	0.0080	5.09E-10	0.6632	-0.0289	0.0024	2.10E-32
HbA1c	9:139328722_G/T	9	139328722	T	G	0.2059	-0.0216	0.0077	5.26E-03	0.4289	-0.0171	0.0023	1.98E-13
HbA1c	9:22130065_A/G	9	22130065	G	A	0.3988	-0.0472	0.0064	1.90E-13	0.0933	0.0421	0.0040	1.48E-26
HbA1c	9:22137685_T/G	9	22137685	G	T	0.3578	0.0257	0.0065	7.89E-05	0.2558	-0.0258	0.0026	1.14E-22
HbA1c	11:2858440_G/A	11	2858440	A	G	0.3892	-0.0573	0.0068	5.29E-17	NA	NA	NA	NA
HbA1c	12:31466613_G/A	12	31466613	A	G	0.1289	0.0435	0.0095	4.18E-06	NA	NA	NA	NA
T2D	1:120517959_G/T	1	120517959	T	G	0.0283	0.1211	0.0457	8.07E-03	0.1100	0.0940	0.0120	4.80E-15
T2D	1:154336716_G/A	1	154336716	A	G	0.1066	0.0747	0.0254	3.26E-03	0.2600	0.0130	0.0085	1.10E-01
T2D	1:207652176_A/G	1	207652176	G	A	0.2665	0.0080	0.0178	6.53E-01	0.0300	0.0300	0.0230	2.00E-01
T2D	1:214154719_G/T	1	214154719	T	G	0.1915	-0.0707	0.0219	1.25E-03	0.3500	-0.0660	0.0079	1.00E-16
T2D	1:214159256_T/C	1	214159256	C	T	0.3657	0.0690	0.0162	2.17E-05	0.5500	0.0710	0.0075	4.80E-21
T2D	1:219753509_T/C	1	219753509	C	T	0.3311	-0.0493	0.0169	3.46E-03	0.2900	-0.0600	0.0082	1.60E-13
T2D	1:233340154_A/G	1	233340154	G	A	0.2571	0.0002	0.0180	9.90E-01	0.0600	-0.0150	0.0160	3.60E-01
T2D	1:241188282_C/T	1	241188282	T	C	0.1550	-0.0343	0.0243	1.57E-01	0.4300	-0.0120	0.0075	1.00E-01
T2D	1:40035928_G/T	1	40035928	T	G	0.1036	0.0668	0.0258	9.61E-03	0.2000	0.0750	0.0094	9.60E-16
T2D	1:50909985_G/A	1	50909985	A	G	0.1113	-0.1406	0.0269	1.78E-07	0.0930	-0.0680	0.0130	1.50E-07
T2D	1:88416590_T/C	1	88416590	C	T	0.1337	-0.0154	0.0241	5.23E-01	0.1000	0.0150	0.0130	2.40E-01
T2D	1:92194322_C/A	1	92194322	A	C	0.4007	0.0161	0.0163	3.22E-01	0.6300	-0.0088	0.0077	2.50E-01
T2D	2:111950541_T/C	2	111950541	C	T	0.5722	-0.0299	0.0163	6.68E-02	0.6400	-0.0270	0.0078	4.40E-04
T2D	2:128421245_G/C	2	128421245	C	G	0.0014	0.3075	0.3038	3.12E-01	0.0180	0.0150	0.0280	5.90E-01
T2D	2:161171454_T/C	2	161171454	C	T	0.8256	0.0188	0.0210	3.69E-01	0.7800	0.0260	0.0091	4.30E-03
T2D	2:165501849_A/C	2	165501849	C	A	0.1344	-0.0183	0.0232	4.30E-01	0.3600	-0.0790	0.0078	6.10E-24
T2D	2:227020653_A/G	2	227020653	G	A	0.1681	0.0238	0.0209	2.56E-01	0.3500	-0.0900	0.0078	1.30E-30
T2D	2:227093585_A/C	2	227093585	C	A	0.9443	0.1049	0.0354	3.05E-03	0.6500	0.1000	0.0078	2.40E-38

T2D	2:27741237_T/C	2	27741237	C	T	0.4575	0.0746	0.0158	2.36E-06	0.6100	0.0680	0.0077	9.20E-19
T2D	2:31068019_C/G	2	31068019	G	C	0.3444	0.0117	0.0168	4.86E-01	0.1600	-0.0120	0.0100	2.40E-01
T2D	2:3841420_T/A	2	3841420	A	T	0.4046	-0.0275	0.0168	1.03E-01	0.3400	-0.0008	0.0079	9.20E-01
T2D	2:43732823_T/C	2	43732823	C	T	0.0052	0.0318	0.1090	7.70E-01	0.1100	-0.1300	0.0120	9.40E-27
T2D	2:53397048_C/G	2	53397048	G	C	0.1573	-0.0009	0.0224	9.68E-01	0.3500	0.0030	0.0078	7.00E-01
T2D	2:57287411_A/G	2	57287411	G	A	0.2336	0.0178	0.0187	3.41E-01	0.5100	0.0180	0.0075	1.80E-02
T2D	2:60557705_A/G	2	60557705	G	A	0.4879	-0.0042	0.0165	8.01E-01	0.4900	-0.0320	0.0075	2.50E-05
T2D	2:60584819_G/A	2	60584819	A	G	0.6604	0.0635	0.0167	1.49E-04	0.4600	0.0580	0.0075	7.60E-15
T2D	2:622827_T/C	2	622827	C	T	0.9081	0.1083	0.0285	1.41E-04	0.8300	0.0081	0.0099	4.10E-01
T2D	3:102203045_C/A	3	102203045	A	C	0.3055	-0.0057	0.0171	7.40E-01	0.0990	0.0070	0.0130	5.80E-01
T2D	3:114850836_C/T	3	114850836	T	C	0.6299	0.0522	0.0171	2.19E-03	0.1100	0.0240	0.0120	4.80E-02
T2D	3:114913508_A/G	3	114913508	G	A	0.6976	0.0407	0.0179	2.33E-02	0.2400	0.0300	0.0088	6.50E-04
T2D	3:123139863_G/A	3	123139863	A	G	0.3605	-0.0518	0.0172	2.54E-03	0.0083	0.0039	0.0430	9.30E-01
T2D	3:12393125_C/G	3	12393125	G	C	0.0497	-0.1258	0.0375	7.90E-04	0.1300	-0.1100	0.0110	1.30E-23
T2D	3:142431000_G/C	3	142431000	C	G	0.0926	-0.0015	0.0288	9.60E-01	0.0430	0.0048	0.0180	8.00E-01
T2D	3:170724883_T/C	3	170724883	C	T	0.2228	-0.0921	0.0196	2.76E-06	0.2900	-0.0790	0.0082	7.30E-22
T2D	3:179661318_G/A	3	179661318	A	G	0.1377	-0.0168	0.0231	4.67E-01	0.3300	0.0048	0.0079	5.40E-01
T2D	3:185529080_A/C	3	185529080	C	A	0.3126	0.1215	0.0168	5.15E-13	0.3200	0.1200	0.0080	1.90E-50
T2D	3:186666461_C/T	3	186666461	T	C	0.1684	-0.0773	0.0215	3.19E-04	0.1400	-0.0660	0.0110	1.80E-09
T2D	3:23077761_T/C	3	23077761	C	T	0.3251	-0.0219	0.0173	2.04E-01	0.4800	0.0008	0.0075	9.20E-01
T2D	3:31176875_A/G	3	31176875	G	A	0.0883	0.0041	0.0292	8.89E-01	0.2500	-0.0160	0.0087	6.40E-02
T2D	3:55313400_A/C	3	55313400	C	A	0.7640	-0.0083	0.0186	6.56E-01	0.8000	-0.0085	0.0093	3.60E-01
T2D	3:64048297_T/C	3	64048297	C	T	0.6288	0.0977	0.0165	3.07E-09	0.8100	0.0350	0.0095	2.10E-04
T2D	3:64705365_C/T	3	64705365	T	C	0.7985	-0.0085	0.0196	6.63E-01	0.4100	-0.0550	0.0076	3.80E-13
T2D	3:64711904_C/T	3	64711904	T	C	0.3924	-0.0138	0.0162	3.93E-01	0.2400	-0.0610	0.0087	2.00E-12
T2D	4:103988899_G/T	4	103988899	T	G	0.3577	0.0291	0.0169	8.47E-02	0.4100	-0.0330	0.0076	1.20E-05
T2D	4:122660250_A/G	4	122660250	G	A	0.0116	-0.0114	0.0858	8.94E-01	0.0500	0.0530	0.0180	3.50E-03
T2D	4:122665514_C/T	4	122665514	T	C	0.3248	0.0154	0.0168	3.60E-01	0.4000	0.0018	0.0076	8.10E-01
T2D	4:129526996_G/C	4	129526996	C	G	0.2192	0.0229	0.0192	2.34E-01	0.0550	0.0530	0.0170	1.30E-03
T2D	4:1309901_C/G	4	1309901	G	C	0.3914	-0.1070	0.0163	5.67E-11	0.0300	-0.0740	0.0240	2.20E-03
T2D	4:153520475_C/T	4	153520475	T	C	0.4651	-0.0861	0.0163	1.40E-07	0.2800	-0.0630	0.0084	7.00E-14
T2D	4:164532801_T/C	4	164532801	C	T	0.1749	-0.0069	0.0208	7.38E-01	0.0200	-0.0110	0.0290	7.10E-01
T2D	4:185708807_T/C	4	185708807	C	T	0.0115	0.0671	0.0826	4.17E-01	0.1700	-0.0560	0.0100	3.90E-08
T2D	4:6270056_G/A	4	6270056	A	G	0.9798	0.1139	0.0580	4.96E-02	0.5800	0.0830	0.0076	1.60E-27
T2D	4:941518_C/T	4	941518	T	C	0.2560	0.0181	0.0180	3.16E-01	0.1500	0.0017	0.0110	8.70E-01
T2D	4:95012684_C/A	4	95012684	A	C	0.8173	-0.0225	0.0211	2.88E-01	0.4700	-0.0250	0.0075	8.60E-04
T2D	5:101620174_T/C	5	101620174	C	T	0.6651	0.0126	0.0171	4.59E-01	0.7400	-0.0440	0.0085	2.30E-07
T2D	5:112809728_C/T	5	112809728	T	C	0.0978	0.0239	0.0269	3.74E-01	0.3300	-0.0083	0.0079	2.90E-01

T2D	5:133864599_G/A	5	133864599	A	G	0.4098	0.0430	0.0161	7.47E-03	0.4200	0.0550	0.0075	2.00E-13
T2D	5:134240235_T/C	5	134240235	C	T	0.6427	0.0272	0.0167	1.04E-01	0.5800	0.0220	0.0076	4.00E-03
T2D	5:53271420_G/A	5	53271420	A	G	0.8916	0.0352	0.0259	1.74E-01	0.6900	0.0600	0.0081	9.70E-14
T2D	5:55806751_A/G	5	55806751	G	A	0.5124	0.1029	0.0158	8.14E-11	0.7400	0.0790	0.0085	1.10E-20
T2D	5:55856375_G/T	5	55856375	T	G	0.0927	0.0397	0.0269	1.40E-01	0.2000	0.0660	0.0093	1.30E-12
T2D	5:56104308_A/G	5	56104308	G	A	0.5729	0.0089	0.0166	5.92E-01	0.8200	-0.0260	0.0098	9.00E-03
T2D	5:76424949_G/A	5	76424949	A	G	0.9753	-0.0649	0.0502	1.96E-01	0.6900	-0.0690	0.0081	1.50E-17
T2D	6:126755642_T/G	6	126755642	G	T	0.9901	0.1675	0.0868	5.37E-02	0.4600	0.0530	0.0075	1.10E-12
T2D	6:126964510_A/G	6	126964510	G	A	0.4726	0.0700	0.0158	1.00E-05	0.2700	0.0690	0.0084	2.60E-16
T2D	6:137287702_A/G	6	137287702	G	A	0.5434	-0.0437	0.0166	8.45E-03	0.4700	-0.0510	0.0076	2.10E-11
T2D	6:140273647_G/A	6	140273647	A	G	0.3794	-0.0116	0.0165	4.83E-01	0.2400	0.0160	0.0088	7.10E-02
T2D	6:160766770_T/C	6	160766770	C	T	0.3510	-0.0399	0.0167	1.69E-02	0.4900	-0.0460	0.0075	8.60E-10
T2D	6:20661250_G/C	6	20661250	C	G	0.4759	0.2833	0.0159	3.11E-71	0.3200	0.1300	0.0079	6.90E-58
T2D	6:31136453_A/G	6	31136453	G	A	0.7242	0.0409	0.0177	2.12E-02	0.7400	0.0560	0.0086	8.70E-11
T2D	6:31347451_A/G	6	31347451	G	A	0.4859	-0.0052	0.0158	7.45E-01	0.3500	0.0180	0.0078	1.90E-02
T2D	6:32339897_C/T	6	32339897	T	C	0.5938	0.0870	0.0161	6.86E-08	0.6700	0.0470	0.0081	7.00E-09
T2D	6:32428115_G/A	6	32428115	A	G	0.3589	0.0706	0.0164	1.59E-05	0.3000	0.0580	0.0083	4.30E-12
T2D	6:32685550_G/A	6	32685550	A	G	0.1550	0.0187	0.0216	3.87E-01	0.1200	0.0910	0.0120	4.60E-15
T2D	6:38106844_T/C	6	38106844	C	T	0.2450	0.0336	0.0193	8.13E-02	0.0900	0.0110	0.0130	4.20E-01
T2D	6:39284050_G/T	6	39284050	T	G	0.4072	0.0539	0.0160	7.37E-04	0.4900	0.0280	0.0074	1.60E-04
T2D	6:43811762_C/T	6	43811762	T	C	0.0922	0.0166	0.0277	5.49E-01	0.2900	0.0530	0.0082	1.00E-10
T2D	6:65533066_C/T	6	65533066	T	C	0.0955	0.0375	0.0270	1.64E-01	0.1900	-0.0013	0.0096	8.90E-01
T2D	6:66618657_G/A	6	66618657	A	G	0.0489	-0.0380	0.0376	3.11E-01	0.3000	0.0068	0.0082	4.10E-01
T2D	6:71289189_A/G	6	71289189	G	A	0.0855	-0.0516	0.0285	7.03E-02	0.1800	0.0005	0.0098	9.60E-01
T2D	6:7258617_C/T	6	7258617	T	C	0.6291	-0.0235	0.0169	1.65E-01	0.2800	-0.0290	0.0083	5.00E-04
T2D	6:7290437_A/G	6	7290437	G	A	0.4659	-0.0133	0.0158	4.01E-01	0.4100	-0.0500	0.0076	4.30E-11
T2D	7:127164958_A/G	7	127164958	G	A	0.7574	0.0652	0.0187	4.94E-04	0.5300	-0.0200	0.0075	7.70E-03
T2D	7:127862802_A/G	7	127862802	G	A	0.8953	-0.1340	0.0253	1.18E-07	0.8200	0.0075	0.0097	4.40E-01
T2D	7:130442141_C/G	7	130442141	G	C	0.4588	0.0457	0.0161	4.52E-03	0.3200	0.0610	0.0081	5.00E-14
T2D	7:130466854_A/G	7	130466854	G	A	0.7062	0.0221	0.0174	2.04E-01	0.5200	0.0560	0.0075	6.70E-14
T2D	7:13900731_A/G	7	13900731	G	A	0.3993	0.0524	0.0161	1.10E-03	0.5100	0.0290	0.0075	8.90E-05
T2D	7:140612163_A/T	7	140612163	T	A	0.1462	0.0199	0.0233	3.92E-01	0.1400	0.0340	0.0110	1.60E-03
T2D	7:14898282_C/T	7	14898282	T	C	0.4108	0.0964	0.0167	8.52E-09	0.1700	0.0730	0.0098	7.50E-14
T2D	7:15064309_G/T	7	15064309	T	G	0.6725	0.1129	0.0169	2.64E-11	0.5400	0.0700	0.0075	5.50E-21
T2D	7:157027753_T/C	7	157027753	C	T	0.9224	0.0643	0.0350	6.60E-02	0.8200	0.0660	0.0098	2.30E-11
T2D	7:28180556_T/C	7	28180556	C	T	0.2692	-0.0761	0.0180	2.33E-05	0.5000	-0.0980	0.0074	4.90E-40
T2D	7:28198677_C/T	7	28198677	T	C	0.0044	-0.0508	0.1255	6.85E-01	0.4900	-0.1000	0.0075	8.70E-41
T2D	7:30714436_A/G	7	30714436	G	A	0.3875	0.0158	0.0163	3.35E-01	0.6300	0.0190	0.0077	1.30E-02

T2D	7:30728452_C/T	7	30728452	T	C	0.1724	-0.0408	0.0215	5.75E-02	0.2300	-0.0440	0.0091	1.40E-06
T2D	7:44235668_G/A	7	44235668	A	G	0.2126	0.0163	0.0192	3.95E-01	0.1700	0.0610	0.0099	5.90E-10
T2D	7:44245363_A/G	7	44245363	G	A	0.3078	-0.0535	0.0177	2.50E-03	0.4300	-0.0390	0.0076	2.90E-07
T2D	8:118184783_C/T	8	118184783	T	C	0.4021	-0.1654	0.0164	5.44E-24	0.3100	-0.1200	0.0081	3.10E-53
T2D	8:12811580_T/G	8	12811580	G	T	0.5768	0.0064	0.0165	6.99E-01	0.2900	0.0023	0.0082	7.80E-01
T2D	8:129568078_C/T	8	129568078	T	C	0.9634	-0.0808	0.0429	5.94E-02	0.7300	-0.0390	0.0084	2.90E-06
T2D	8:145536056_A/G	8	145536056	G	A	0.4840	0.0825	0.0161	3.20E-07	0.3800	0.0600	0.0078	1.80E-14
T2D	8:36859186_T/C	8	36859186	C	T	0.3601	0.0468	0.0168	5.20E-03	0.1700	0.0350	0.0100	4.50E-04
T2D	8:41509259_G/A	8	41509259	A	G	0.4274	0.0848	0.0160	1.16E-07	0.5800	0.0700	0.0076	2.00E-20
T2D	8:41519248_T/C	8	41519248	C	T	0.8683	0.1252	0.0241	1.93E-07	0.7600	0.0970	0.0088	5.60E-28
T2D	8:68613390_T/C	8	68613390	C	T	0.1456	0.0232	0.0229	3.10E-01	0.0800	-0.0018	0.0140	9.00E-01
T2D	8:95856911_A/G	8	95856911	G	A	0.0440	0.0231	0.0387	5.50E-01	0.1900	0.0310	0.0095	1.20E-03
T2D	8:95960511_T/C	8	95960511	C	T	0.7038	-0.0431	0.0172	1.24E-02	0.5000	-0.0510	0.0074	6.10E-12
T2D	9:112526289_C/G	9	112526289	G	C	0.2484	0.0036	0.0187	8.47E-01	0.1800	0.0001	0.0099	9.90E-01
T2D	9:136155000_C/T	9	136155000	T	C	0.2589	0.0691	0.0177	9.68E-05	0.1900	0.0430	0.0094	5.60E-06
T2D	9:139252148_G/A	9	139252148	A	G	0.9351	0.1697	0.0363	2.86E-06	0.6600	0.0480	0.0080	2.70E-09
T2D	9:22051670_G/C	9	22051670	C	G	0.8613	-0.0173	0.0230	4.51E-01	0.5700	0.0580	0.0075	7.60E-15
T2D	9:22134094_T/C	9	22134094	C	T	0.4415	-0.2467	0.0161	1.00E-52	0.1700	-0.1800	0.0100	7.10E-69
T2D	9:22137685_T/G	9	22137685	G	T	0.3554	0.0541	0.0165	1.07E-03	0.2600	0.1200	0.0085	4.10E-45
T2D	9:22301092_T/A	9	22301092	A	T	0.0425	-0.1505	0.0430	4.60E-04	0.0330	-0.0960	0.0210	6.40E-06
T2D	9:28772700_A/T	9	28772700	T	A	0.3753	0.0050	0.0167	7.63E-01	0.2400	0.0100	0.0088	2.60E-01
T2D	9:4287466_A/G	9	4287466	G	A	0.5510	-0.0896	0.0158	1.55E-08	0.5000	-0.0320	0.0074	1.70E-05
T2D	9:4297892_T/C	9	4297892	C	T	0.3339	-0.0632	0.0176	3.42E-04	0.2800	-0.0440	0.0085	1.60E-07
T2D	9:81952128_C/T	9	81952128	T	C	0.1095	-0.1293	0.0262	7.83E-07	0.0720	-0.0910	0.0150	5.70E-10
T2D	9:84308948_G/A	9	84308948	A	G	0.5878	-0.0766	0.0164	3.05E-06	0.4100	-0.0800	0.0076	1.30E-25
T2D	9:93633240_A/C	9	93633240	C	A	0.2208	-0.0084	0.0190	6.58E-01	0.0100	0.0018	0.0430	9.70E-01
T2D	9:94395860_A/T	9	94395860	T	A	0.0719	0.0015	0.0307	9.62E-01	0.1300	0.0170	0.0110	1.10E-01
T2D	10:114758349_C/T	10	114758349	T	C	0.0284	0.2972	0.0434	7.31E-12	0.2900	0.3600	0.0082	0.00E+00
T2D	10:121149403_C/T	10	121149403	T	C	0.2196	-0.0072	0.0190	7.04E-01	0.4800	-0.0089	0.0074	2.30E-01
T2D	10:122849667_A/G	10	122849667	G	A	0.2210	-0.0428	0.0201	3.31E-02	0.1700	-0.0230	0.0100	2.10E-02
T2D	10:12307894_C/T	10	12307894	T	C	0.4515	0.1439	0.0158	6.52E-20	0.2100	0.0950	0.0090	7.70E-26
T2D	10:12328010_A/G	10	12328010	G	A	0.1316	0.1128	0.0237	1.87E-06	0.1900	0.0910	0.0096	2.10E-21
T2D	10:124186714_C/T	10	124186714	T	C	0.6398	-0.0304	0.0167	6.91E-02	0.4800	-0.0470	0.0075	4.00E-10
T2D	10:132947962_C/G	10	132947962	G	C	0.8615	0.0126	0.0229	5.82E-01	0.9270	0.0130	0.0140	3.60E-01
T2D	10:65101071_G/C	10	65101071	C	G	0.6255	-0.0336	0.0162	3.85E-02	0.8600	0.0200	0.0110	5.70E-02
T2D	10:70931474_C/T	10	70931474	T	C	0.1178	-0.0783	0.0249	1.63E-03	0.3100	-0.0006	0.0080	9.40E-01
T2D	10:71452285_T/C	10	71452285	C	T	0.9267	-0.0379	0.0325	2.44E-01	0.8300	-0.0600	0.0100	2.50E-09
T2D	10:71465928_A/G	10	71465928	G	A	0.9671	-0.0312	0.0442	4.81E-01	0.7800	0.0470	0.0091	1.90E-07

T2D	10:80942631_A/G	10	80942631	G	A	0.4460	-0.0433	0.0162	7.64E-03	0.4600	-0.0790	0.0075	1.10E-25
T2D	10:89768584_G/A	10	89768584	A	G	0.3661	0.0393	0.0164	1.69E-02	0.1500	0.0570	0.0100	5.30E-08
T2D	10:94462882_C/T	10	94462882	T	C	0.6886	-0.1609	0.0168	7.75E-22	0.4100	-0.1200	0.0076	1.30E-57
T2D	10:94658207_G/A	10	94658207	A	G	0.0409	0.2880	0.0380	3.54E-14	0.1800	0.0290	0.0096	2.10E-03
T2D	10:94658207_G/A	10	94658207	A	G	0.0409	0.2880	0.0380	3.54E-14	0.1800	-0.0340	0.0290	2.40E-01
T2D	11:17408630_C/T	11	17408630	T	C	0.6056	-0.1048	0.0161	6.71E-11	0.6300	-0.0790	0.0077	9.40E-25
T2D	11:2161530_C/T	11	2161530	T	C	0.0188	0.0745	0.0574	1.94E-01	0.0005	0.4900	0.3600	1.80E-01
T2D	11:2215089_G/A	11	2215089	A	G	0.9134	-0.0738	0.0277	7.77E-03	0.7100	-0.0350	0.0083	2.80E-05
T2D	11:2691471_A/G	11	2691471	G	A	0.8764	0.0756	0.0245	2.07E-03	0.5200	0.0640	0.0076	2.40E-17
T2D	11:2858440_G/A	11	2858440	A	G	0.3857	-0.3081	0.0182	2.60E-64	0.0440	-0.2200	0.0190	1.90E-29
T2D	11:41915366_C/A	11	41915366	A	C	0.2249	-0.0369	0.0198	6.25E-02	0.0890	-0.0150	0.0130	2.50E-01
T2D	11:43877934_C/A	11	43877934	A	C	0.1931	0.0254	0.0199	2.02E-01	0.2900	0.0320	0.0082	7.80E-05
T2D	11:72433098_A/C	11	72433098	C	A	0.0624	-0.1895	0.0348	5.36E-08	0.1600	-0.1300	0.0100	3.70E-35
T2D	11:92708710_C/G	11	92708710	G	C	0.4259	0.1331	0.0158	4.06E-17	0.2800	0.1000	0.0083	2.80E-36
T2D	12:121402932_C/T	12	121402932	T	C	0.4712	0.0124	0.0158	4.34E-01	0.2300	-0.0470	0.0089	1.10E-07
T2D	12:121432299_C/T	12	121432299	T	C	0.1624	0.1329	0.0213	4.41E-10	0.0160	-0.0650	0.0300	3.30E-02
T2D	12:121686929_G/A	12	121686929	A	G	0.3351	-0.0038	0.0172	8.27E-01	0.3100	-0.0280	0.0081	5.20E-04
T2D	12:123447928_T/C	12	123447928	C	T	0.6501	0.0401	0.0170	1.85E-02	0.7100	0.0410	0.0083	7.80E-07
T2D	12:123640853_C/G	12	123640853	G	C	0.9703	0.0212	0.0490	6.65E-01	0.8000	0.0500	0.0093	7.20E-08
T2D	12:21752108_G/T	12	21752108	T	G	0.3430	0.0060	0.0178	7.36E-01	0.2400	-0.0330	0.0090	2.30E-04
T2D	12:26457650_A/T	12	26457650	T	A	0.5703	0.0318	0.0166	5.49E-02	0.2300	0.0600	0.0089	1.50E-11
T2D	12:27958113_C/T	12	27958113	T	C	0.1918	0.0763	0.0210	2.83E-04	0.4800	0.0360	0.0076	1.90E-06
T2D	12:27965150_C/T	12	27965150	T	C	0.2112	-0.0588	0.0200	3.22E-03	0.2000	-0.0770	0.0094	2.00E-16
T2D	12:31466613_G/A	12	31466613	A	G	0.1294	0.1875	0.0236	2.00E-15	0.0097	0.1300	0.1100	2.30E-01
T2D	12:4374373_A/G	12	4374373	G	A	0.0624	0.0738	0.0390	5.83E-02	0.2100	0.0690	0.0092	8.20E-14
T2D	12:51357542_A/G	12	51357542	G	A	0.5103	-0.0097	0.0158	5.40E-01	0.1600	-0.0120	0.0100	2.30E-01
T2D	12:55098996_T/A	12	55098996	A	T	0.9786	0.0068	0.0549	9.02E-01	0.7400	0.0074	0.0085	3.80E-01
T2D	12:66212318_C/T	12	66212318	T	C	0.1217	0.0652	0.0245	7.84E-03	0.0970	0.1200	0.0130	2.80E-21
T2D	12:66250940_T/G	12	66250940	G	T	0.6583	-0.0778	0.0166	2.72E-06	0.8600	-0.0910	0.0110	2.00E-17
T2D	12:66379504_T/G	12	66379504	G	T	0.8518	0.0371	0.0242	1.26E-01	0.3200	0.0450	0.0081	2.10E-08
T2D	12:71433293_C/T	12	71433293	T	C	0.3085	0.0186	0.0174	2.86E-01	0.5500	-0.0450	0.0075	2.70E-09
T2D	12:71663102_C/T	12	71663102	T	C	0.7690	-0.0501	0.0186	7.01E-03	0.7200	-0.0400	0.0083	1.50E-06
T2D	12:77398721_A/T	12	77398721	T	A	0.1083	-0.0388	0.0259	1.34E-01	0.1800	-0.0130	0.0098	1.90E-01
T2D	13:26781528_G/A	13	26781528	A	G	0.4471	-0.0739	0.0160	4.11E-06	0.2300	-0.0480	0.0089	7.40E-08
T2D	13:33554302_G/A	13	33554302	A	G	0.8353	-0.0930	0.0210	9.16E-06	0.8300	-0.0530	0.0100	1.30E-07
T2D	13:80717156_G/A	13	80717156	A	G	0.3002	-0.1222	0.0176	3.51E-12	0.2800	-0.0910	0.0083	6.50E-28
T2D	13:91940169_T/A	13	91940169	A	T	0.1480	-0.1074	0.0232	3.69E-06	0.2600	-0.0330	0.0086	1.20E-04
T2D	14:101142890_G/A	14	101142890	A	G	0.1068	-0.0068	0.0257	7.92E-01	0.8600	-0.0310	0.0110	4.90E-03

T2D	15:38822905_T/C	15	38822905	C	T	0.5662	-0.0385	0.0159	1.56E-02	0.7800	-0.0320	0.0089	2.70E-04
T2D	15:38822905_T/C	15	38822905	C	T	0.5662	-0.0385	0.0159	1.56E-02	0.7800	-0.1500	0.0970	1.20E-01
T2D	15:40619724_T/C	15	40619724	C	T	0.1753	0.1079	0.0205	1.46E-07	0.1100	0.0350	0.0120	4.00E-03
T2D	15:62396389_A/G	15	62396389	G	A	0.4629	-0.0938	0.0158	3.10E-09	0.4300	-0.0470	0.0075	5.20E-10
T2D	15:77747190_A/G	15	77747190	G	A	0.4234	0.0892	0.0159	1.96E-08	0.7100	0.0710	0.0082	4.30E-18
T2D	15:80432222_A/G	15	80432222	G	A	0.0884	0.0545	0.0272	4.54E-02	0.6600	0.0290	0.0079	2.20E-04
T2D	15:90374257_C/A	15	90374257	A	C	0.8125	-0.0722	0.0198	2.68E-04	0.7200	-0.0750	0.0083	1.50E-19
T2D	15:91505779_G/C	15	91505779	C	G	0.5107	0.0939	0.0167	2.00E-08	0.1400	0.0690	0.0110	1.50E-10
T2D	15:91544076_A/G	15	91544076	G	A	0.9793	0.0517	0.0568	3.63E-01	0.3100	0.0440	0.0081	5.60E-08
T2D	16:300641_G/A	16	300641	A	G	0.4035	-0.0421	0.0162	9.41E-03	0.1800	-0.0620	0.0099	4.60E-10
T2D	16:53816275_C/A	16	53816275	A	C	0.1240	0.1162	0.0230	4.30E-07	0.4000	0.0520	0.0076	1.10E-11
T2D	16:75245937_C/T	16	75245937	T	C	0.0248	-0.1074	0.0563	5.67E-02	0.1000	-0.1300	0.0120	1.20E-25
T2D	16:75247245_T/G	16	75247245	G	T	0.1901	0.0224	0.0201	2.65E-01	0.1000	-0.1200	0.0130	1.40E-21
T2D	16:79406918_C/T	16	79406918	T	C	0.2041	0.0102	0.0196	6.04E-01	0.0160	0.0640	0.0390	9.80E-02
T2D	16:81534790_T/C	16	81534790	C	T	0.6329	-0.0468	0.0163	4.02E-03	0.7000	-0.0480	0.0081	2.50E-09
T2D	16:85735652_A/G	16	85735652	G	A	0.3198	-0.0042	0.0178	8.15E-01	0.6300	-0.0270	0.0078	5.40E-04
T2D	17:17654319_A/G	17	17654319	G	A	0.1478	-0.0203	0.0222	3.60E-01	0.6800	-0.0440	0.0080	4.70E-08
T2D	17:27647630_G/T	17	27647630	T	G	0.8451	0.0271	0.0220	2.19E-01	0.7400	-0.0270	0.0086	1.60E-03
T2D	17:36098040_G/A	17	36098040	A	G	0.6726	-0.1451	0.0166	2.36E-18	0.5200	-0.0820	0.0075	5.90E-28
T2D	17:4014384_G/T	17	4014384	T	G	0.2317	-0.0038	0.0218	8.60E-01	0.3100	0.0510	0.0080	2.00E-10
T2D	17:46994970_G/A	17	46994970	A	G	0.7282	0.0210	0.0178	2.38E-01	0.5300	0.0330	0.0075	1.20E-05
T2D	17:47000251_C/T	17	47000251	T	C	0.7473	0.0229	0.0183	2.11E-01	0.5700	0.0280	0.0075	2.30E-04
T2D	17:48636534_G/A	17	48636534	A	G	0.0749	-0.0927	0.0315	3.22E-03	0.3600	-0.0320	0.0078	3.30E-05
T2D	17:6945087_G/T	17	6945087	T	G	0.1006	0.0959	0.0255	1.72E-04	0.0081	0.0600	0.0440	1.70E-01
T2D	18:21839152_A/T	18	21839152	T	A	0.0349	0.0252	0.0446	5.72E-01	0.1100	0.0210	0.0120	8.70E-02
T2D	18:2948029_C/T	18	2948029	T	C	0.4976	0.0013	0.0158	9.33E-01	0.1100	0.0180	0.0120	1.30E-01
T2D	18:40772286_G/C	18	40772286	C	G	0.1251	-0.0108	0.0259	6.77E-01	0.5100	0.0150	0.0075	5.00E-02
T2D	18:57884750_G/A	18	57884750	A	G	0.1951	0.0688	0.0196	4.37E-04	0.2600	0.0130	0.0084	1.10E-01
T2D	18:60845884_T/C	18	60845884	C	T	0.4630	-0.0598	0.0160	1.81E-04	0.3800	-0.0700	0.0079	5.60E-19
T2D	18:7068462_C/G	18	7068462	G	C	0.6688	0.0377	0.0181	3.72E-02	0.3800	0.0380	0.0077	6.10E-07
T2D	19:19610596_C/T	19	19610596	T	C	0.0935	0.0173	0.0277	5.34E-01	0.0840	0.1000	0.0130	1.00E-14
T2D	19:33893008_A/G	19	33893008	G	A	0.4494	-0.0717	0.0160	6.99E-06	0.4200	-0.0400	0.0076	1.60E-07
T2D	19:39580737_A/G	19	39580737	G	A	0.3190	0.0106	0.0170	5.30E-01	0.2100	0.0036	0.0091	6.90E-01
T2D	19:45392254_C/T	19	45392254	T	C	0.0949	-0.0131	0.0273	6.32E-01	0.1700	-0.0400	0.0100	7.50E-05
T2D	19:46172278_G/A	19	46172278	A	G	0.5268	0.1105	0.0161	6.67E-12	0.1100	0.1000	0.0120	6.20E-18
T2D	20:42946966_T/G	20	42946966	G	T	0.5164	0.0788	0.0164	1.66E-06	0.1700	0.0520	0.0100	2.10E-07
T2D	20:45757655_C/T	20	45757655	T	C	0.3980	0.0163	0.0168	3.35E-01	0.5500	0.0031	0.0075	6.80E-01
T2D	21:33385186_A/G	21	33385186	G	A	0.4997	0.0090	0.0158	5.67E-01	0.6200	0.0022	0.0077	7.80E-01

T2D	22:50440296_T/C	22	50440296	C	T	0.5003	0.0622	0.0161	1.10E-04	0.5000	0.0440	0.0075	6.10E-09
T2D	11:65364385_T/A	11	65364385	A	T	0.1600	0.0204	0.0214	3.42E-01	NA	NA	NA	NA
T2D	5:53307054_G/GAATTC	5	53307054	GAATTC	G	0.4727	0.0140	0.0160	3.81E-01	NA	NA	NA	NA
T2D	10:94812254_A/C	10	94812254	C	A	0.0388	0.2669	0.0377	1.55E-12	NA	NA	NA	NA
T2D	12:71611896_AT/ATT	12	71611896	ATT	AT	0.0691	0.0021	0.0345	9.51E-01	NA	NA	NA	NA
T2D	8:82343438_A/G	8	82343438	G	A	0.5069	-0.0362	0.0190	5.67E-02	NA	NA	NA	NA
T2D	7:43320594_C/T	7	43320594	T	C	0.2017	0.0119	0.0197	5.48E-01	NA	NA	NA	NA
T2D	3:23454565_A/G	3	23454565	G	A	0.1954	-0.1488	0.0208	7.96E-13	NA	NA	NA	NA
